# Delay of Reinforcement Versus Rate of Reinforcement in Pavlovian Conditioning

**DOI:** 10.1037/xan0000199

**Published:** 2019-03-07

**Authors:** Joseph M. Austen, David J. Sanderson

**Affiliations:** 1 Department of Psychology, Durham University

**Keywords:** learning, Pavlovian conditioning, timing, mice, reinforcement

## Abstract

Conditioned stimulus (CS) duration is a determinant of conditioned responding, with increases in duration leading to reductions in response rates. The CS duration effect has been proposed to reflect sensitivity to the reinforcement rate across cumulative exposure to the CS, suggesting that the delay of reinforcement from the onset of the cue is not crucial. Here, we compared the effects of delay and rate of reinforcement on Pavlovian appetitive conditioning in mice. In Experiment 1, the influence of reinforcement delay on the timing of responding was removed by making the duration of cues variable across trials. Mice trained with variable duration cues were sensitive to differences in the rate of reinforcement to a similar extent as mice trained with fixed duration cues. Experiments 2 and 3 tested the independent effects of delay and reinforcement rate. In Experiment 2, food was presented at either the termination of the CS or during the CS. In Experiment 3, food occurred during the CS for all cues. The latter experiment demonstrated an effect of delay, but not reinforcement rate. Experiment 4 ruled out the possibility that the lack of effect of reinforcement rate in Experiment 3 was due to mice failing to learn about the nonreinforced CS exposure after the presentation of food within a trial. These results demonstrate that although the CS duration effect is not simply a consequence of timing of conditioned responses, it is dependent on the delay of reinforcement. The results provide a challenge to current associative and nonassociative, time-accumulation models of learning.

Temporal factors play a crucial role in determining the rate of conditioned responding in Pavlovian procedures. One factor is the duration of the conditioned stimulus (CS). Short duration CSs typically elicit higher response rates than long duration CSs (e.g., Gibbon, Baldock, Locurto, Gold, & Terrace, 1977; Harris & Carpenter, 2011; Holland, 2000; Lattal, 1999; but see Davis, Schlesinger, & Sorenson, 1989). An account of this CS duration effect is that it reflects the sensitivity of conditioned responding to the rate of reinforcement across cumulative exposure to a CS (Gallistel & Gibbon, 2000). Recent support for the role of reinforcement rate in the CS duration effect has come from experiments demonstrating no significant difference in the rate of responding elicited by CSs that differ in duration if they are matched for cumulative reinforcement rate (Harris, Patterson, & Gharaei, 2015). Matched reinforcement rate was achieved by reinforcing the short duration CS on only a proportion of trials such that its cumulative reinforcement rate was the same as the long duration CS. For example, in the second experiment by Harris et al. (2015) rats were trained with a CS that was on average 30 s long across trials and another CS that was on average 10 s long. The 30-s CS was reinforced on every trial. The 10-s CS was presented three times as often as the 30-s CS, thereby matching cumulative exposure between the CSs, and was reinforced on a random third of trials. Because of the differences in average CS duration and probability of the unconditioned stimulus (US) per trial, both CSs were reinforced on average every 30 s across cumulative exposure to the CS. The rate of responding to the short- and long-duration CSs did not differ over training. In contrast, conditioned responding was greater for a variable duration 10-s CS compared to a variable duration 30-s CS when both cues were reinforced every trial. Therefore, the advantage of the short-duration CS over the long-duration CS was removed by matching the rate of reinforcement over cumulative exposure. We have also found similar results with mice (Austen, Pickering, Sprengel, & Sanderson, 2018). Using procedures that were similar to Harris et al. (2015), mice received appetitive Pavlovian conditioning of magazine approach behavior. The rate of responding elicited by a 10-s CS did not differ significantly from a 40-s CS when the 10-s CS was reinforced on a random 25% of trials and was presented four times as often as the 40-s CS, thereby matching the number of CS–US pairings. In contrast, when both CSs were reinforced on 100% of trials the 10-s CS elicited a higher rate of responding than the 40-s CS.

In a continuously reinforced delay conditioning procedure in which the US is presented at the termination of the CS on every trial the reinforcement rate is the reciprocal of the delay of reinforcement (CS–US interval). Therefore, under such conditions it is not possible to isolate the role of reinforcement rate from the role of delay of reinforcement. The experiments described above, however, demonstrated an independent role of reinforcement rate by comparing partially reinforced cues with a short delay of reinforcement with continuously reinforced cues with a longer delay of reinforcement such that cumulative reinforcement rate was matched. This suggests that when rate of reinforcement is controlled, delay of reinforcement does not determine the rate of responding. Thus, it does not matter if reinforcement occurs after a short or long delay. One problem with this conclusion is that cues that were matched for reinforcement rate differed in probability of reinforcement per trial as well as delay of reinforcement. One way of disentangling the role of reinforcement rate from the delay of reinforcement that avoids confounding the delay of reinforcement with probability of reinforcement per trial is to compare cues of equal duration that differ in delay of reinforcement by virtue of reinforcement occurring at different time points within the trial. Therefore, reinforcement rate is matched for cues with different CS–US intervals by manipulating the duration of continued exposure to the CS after the US. If conditioned responding is determined by the reinforcement rate over cumulative exposure to the CS regardless of how the exposure is structured relative to the presentation of the US, then it would be predicted that delay of reinforcement will have little effect on response rates.

The prediction that delay of reinforcement will not have an effect on conditioned responding is at odds with other work that does suggest that delay of reinforcement does influence behavior. The delay of reinforcement is known to influence conditioned responding, affecting the distribution of responding within a trial such that responses are timed to the occurrence of reinforcement (e.g., Delamater, Chen, Nasser, & Elayouby, 2018). Based, in part, on the timing of conditioned responding that animals show, it has been proposed that the associative strength of a CS reflects the estimated delay of reinforcement from CS onset (Balsam, 1984; Gibbon & Balsam, 1981). Moreover, there are a number of reports from studies examining choice behavior in appetitive operant conditioning, primarily in pigeons, but also in rats, that the delay of reinforcement has a greater effect than the rate of reinforcement (Davison, 1968; Gentry & Marr, 1980; Herrnstein, 1964; Killeen, 1968; Lea, 1979; Logan, 1965; Mazur, Snyderman, & Coe, 1985; McDiarmid & Rilling, 1965; Shull, Spear, & Bryson, 1981). Thus, responses that lead to a short delay of reinforcement, but with a low rate of reinforcement are preferred to responses that lead to a long delay of reinforcement, but with a high rate of reinforcement.

A further reason for suggesting that the delay of reinforcement is important is that Harris et al. (2015) found that the CS duration effect was abolished by matching reinforcement rates only when the CSs were of variable durations that changed trial by trial such that rats could not time the occurrence of the US within the trial. Specifically, CS durations varied uniformly around a mean duration, which led to rats displaying a flat response rate within a trial. When cues were of a fixed duration, and, therefore, reinforcement occurred after a fixed delay, rats showed faster acquisition of conditioned responding with the long duration cue that was reinforced on every trial compared to the short duration cue that was reinforced on a proportion of trials. Although this may suggest the probability of reinforcement per trial may also play a role in determining response rates in some circumstances (see also Chan & Harris, 2017; Harris & Andrew, 2017; Harris & Kwok, 2018), it demonstrates that timing of conditioned responding may confound measures of overall response rates, masking the relationship between reinforcement rates and response rates.

In contrast to Harris et al. (2015) we found that fixed duration CSs, for which the occurrence of reinforcement could be timed, failed to elicit different response rates when they were matched for reinforcement rate over trials (Austen et al., 2018). The difference between our results with mice and those of Harris et al. with rats may be due to a number of reasons. Regardless of these differences, although the collective results suggest that reinforcement rate is an important determinant of response rates, these experiments confounded CS duration (whether variable or fixed) and consequently delay of reinforcement with probability of reinforcement per trial. Therefore, it is not clear whether delay of reinforcement plays a role in determining overall response rates in Pavlovian conditioning that is independent from reinforcement rate.

Identifying the factors that determine the strength of conditioned responding is crucial for assessing the merits of theoretical accounts of learning. Associative theories that have been developed to explain sensitivity to temporal information (e.g., Ludvig, Sutton, & Kehoe, 2012; Sutton & Barto, 1981; Vogel, Brandon, & Wagner, 2003; Wagner, 1981) assume that both rate and delay of reinforcement will influence the strength of responding, but do not necessarily anticipate that the factors will be dissociable without making assumptions about a number of free parameters. Theories that are explicitly nonassociative, in that they assume learning requires symbolic encoding of quantitative variables, do make claims about which variables are crucial. For example, scalar expectancy theory proposes that responding reflects expectancy of reinforcement based on the comparison of time since the onset of the CS and the remembered CS–US interval (Gibbon, 1977). In contrast, rate estimation theory proposes that conditioned responding reflects comparison of the reinforcement rate of a CS with the background reinforcement rate over cumulative exposure (Gallistel & Gibbon, 2000).

The purpose of the experiments presented here was to assess the role of delay of reinforcement in determining response rates in Pavlovian conditioning in mice. Similar to the study by Austen et al. (2018), appetitive conditioning of magazine approach behavior was used with the rate of head entries into the food magazine as the measure of responding. In Experiment 1, we assessed whether the ability to time the occurrence of the US affected the CS duration effect. This was achieved by comparing the CS duration effect in mice that were trained with short and long duration CSs that were of either a fixed or a variable duration. Experiments 2 and 3 tested the effects of delay of reinforcement and rate of reinforcement in order to determine whether they had independent effects. The effects of delay were assessed by comparing CSs of the same duration but which differed in delay of reinforcement due to manipulation of the time within a trial at which the US was presented. The independent effect of reinforcement rate was assessed by comparing CSs of different durations, but that were reinforced at the same time point from the onset of the CS. Experiment 4 tested an account of the results of Experiment 3 in which, within a trial, continued nonreinforced exposure to a CS after the US failed to reduce response rates.

## Experiment 1

The aim of Experiment 1 was to test the extent to which timing of conditioned responses influences the CS duration effect. Although CSs of different durations that are reinforced at the end of their presentation elicit different overall rates of responding, they also elicit distinct patterns of responding, with responses being timed to the presentation of reinforcement. Therefore, any comparison of overall response rates between cues of different durations is potentially confounded by differences in the distribution of responding across cues. Indeed, Harris et al. (2015) have argued that the sensitivity of response rates to reinforcement rates can only be detected under conditions in which animals are unable to time their responses due to the time of reinforcement being variable. In contrast, we have used fixed duration cues and have failed to find any difference in acquisition with cues of different durations and probability of reinforcement per trial, but that have the same cumulative reinforcement rate (Austen et al., 2018). This was true regardless of whether responding was compared across equivalent numbers of trials, or equivalent numbers of reinforcements. This may suggest that the cue duration effect that we have observed in mice, using continuously reinforced, fixed duration 10-s and 40-s cues, may solely reflect differences in reinforcement rate rather than timing of conditioned responding. Thus, it is possible that mice failed to time responses sufficiently for it to affect overall response rates.

**Figure 1 fig1:**
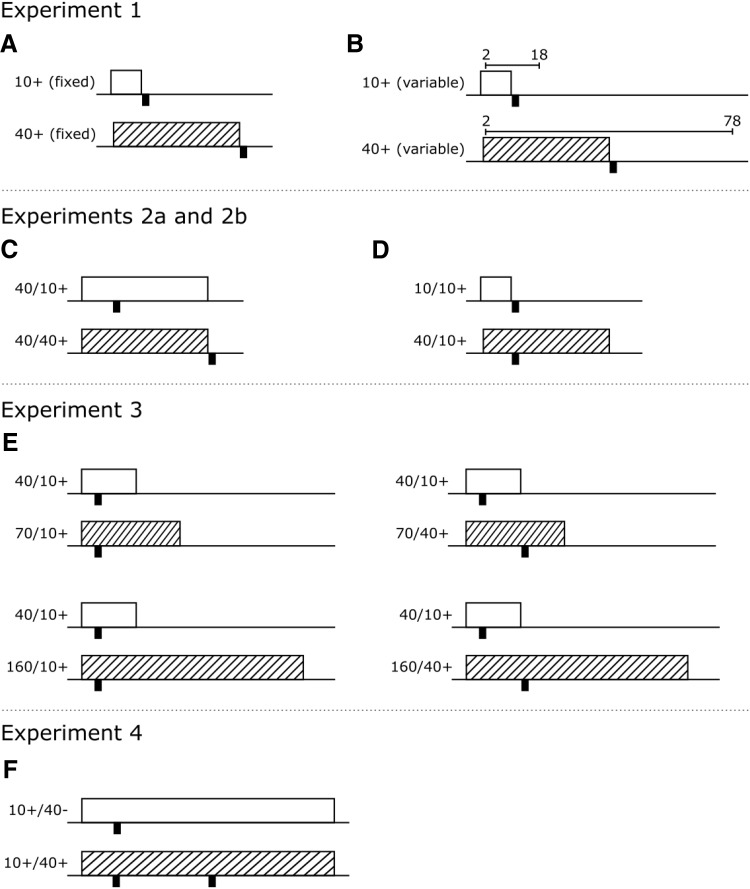
Design of Experiments 1–4. Open and striped rectangles represent cues and their durations. Filled black rectangles represent the presentation of the sucrose reward. (A) Experiment 1: Group fixed; (B) Experiment 1: Group variable; (C) Experiment 2a; (D) Experiment 2b; (E) Experiment 3 (the cue durations are not to scale with the other panels in order to accommodate the 160 s cue); (F) Experiment 4.

To test whether timing of conditioned responding influences the extent of the CS duration effect we trained one group of mice with fixed duration 10-s and 40-s CSs, replicating the design used by Austen et al. (2018), and another group with CSs that varied in duration trial by trial, but one CS was on average 10-s and the other 40-s on average (see Figure 1A and 1B). Harris, Gharaei, and Pincham (2011) have shown that, in rats, when CS durations vary in a uniformly distributed manner, then response rates are constant across the duration of the CS. In contrast, when variable CS durations are drawn from an exponential distribution, response rates decline across the duration of the CS. Therefore, we chose to sample variable cue durations from a uniform distribution, to reduce the likelihood that responding would change as a function of the CS duration and, as a consequence, appear to be timed. In addition to the reinforced CSs, mice also received presentations of nonreinforced (fixed or variable duration, according to group allocation) 10-s and 40-s CSs. These nonreinforced cues served as control cues for determining baseline response rates for the cues of different durations.[Fig-anchor fig1]

### Method

#### Subjects

Thirty-two experimentally naïve female C57BL/6J mice (Charles River UK Ltd., Margate, United Kingdom), approximately 10 weeks old at the start of testing, with a mean free-feeding weight of 19.1*g* (range: 15.9–22.6*g*), were used. Mice were caged in groups of 4–8 in a temperature-controlled housing room on a 12-hr light–dark cycle (lights on at 8:00 a.m.). Prior to the start of the experiment, the weights of the mice were reduced by being placed on a restricted diet. Mice were then maintained at 85% of their free-feeding weights throughout the experiment. Mice had ad libitum access to water in their home cages. All procedures were conducted under Home Office UK project license number PPL 70/7785.

#### Apparatus

A set of eight identical operant chambers (interior dimensions: 15.9 × 14.0 × 12.7 cm; ENV-307A, Med Associates, Inc., Fairfax, VA), enclosed in sound-attenuating cubicles (ENV-022V) were used. The operant chambers were controlled by Med-PC IV software (SOF-735). The side walls were made from aluminum, and the front and back walls and the ceiling were made from clear Perspex. The chamber floors each comprised a grid of stainless steel rods (0.32-cm diameter), spaced 0.79 cm apart, and running perpendicular to the front of the chamber (ENV-307A-GFW). A food magazine (2.9 × 2.5 × 1.9 cm; ENV-303M) was situated in the center of one of the sidewalls of the chamber, into which sucrose pellets (14 mg, TestDiet) could be delivered from a pellet dispenser (ENV-203-14P). An infrared beam (ENV-303HDA) across the entrance of the magazine was used to record head entries at a resolution of 0.1 s. A fan (ENV-025F) was located within each of the sound-attenuating cubicles and was turned on during sessions, providing a background SPL of approximately 65 dB. Auditory stimuli were provided by a white noise generator (ENV-325SM) outputting a flat frequency response from 10 to 25,000 Hz at 75 dB and a clicker (ENV-335M) operating at a frequency of 4 Hz at 75 dB. Visual stimuli were a 2.8 W house light (ENV-315M), which could illuminate the entire chamber, and two LEDs (ENV-321M) positioned to the left and right of the food magazine, which provided more localized illumination.

#### Procedure

Mice received 12 sessions of training with two short duration cues and two long duration cues. Mice were randomly allocated to one of two groups (*N* = 16 per group). For group fixed, the duration of short cues was 10 s and the duration of long cues was 40 s. For group variable, the durations of the cues varied from trial to trial, but within a session they had a mean duration that was the same as the duration of group fixed. Therefore, the short cues had a mean of 10 s, but the duration of each trial varied, according to a uniform distribution, around the mean (shortest = 2 s, longest = 18 s). Similarly, the long cues had a mean of 40 s, and trials varied according to a uniform distribution around the mean (shortest = 2 s, longest = 78 s). For both groups one of the short and one of the long duration cues was reinforced by presentation of a sucrose pellet at the termination of the cue (CS+). The remaining short and long cues were nonreinforced (CS−). Within each group, for half of the mice the short cues were auditory (noise, clicker) and the long cues were visual (house light, flashing LEDs [0.25 s on/0.25 s off]). The opposite was true for the remaining mice. Within each of these subgroups the identity of the reinforced and nonreinforced stimuli was fully counterbalanced. Each of the four cues was presented nine times per session with a fixed interval of 120 s between the offset of one cue and the onset of the next. Trials were presented in a random order with the constraint that an equal number of each cue was presented every block of 12 trials. For each session, all mice received the stimuli presented in the same order (e.g., 1st trial = noise, 2nd trial = house light, 3rd trial = clicker, etc.). Because the identity of short and long duration cues and the identity of reinforced and nonreinforced cues were counterbalanced across mice, this resulted in the order of these factors also being counterbalanced across mice.

#### Data and statistical analysis

The frequency of head entries into the food magazine was recorded per-second during the CS presentations and for the 10-s pre-CS period. Responding during the CS is reported as a difference score (responses per minute [RPM]) in which responding during the nonreinforced cues was subtracted from the responding to the reinforced cue of the same duration and modality. For mice that were trained with variable duration cues, the overall response rates were calculated by averaging across response rates per trial independent of the duration of the trial.

Timing of conditioned responding was analyzed by calculating the mean response rate per 1-s time bin for individual mice. For mice trained with variable duration cues the response rate per 1-s time bin was calculated by averaging over the response rates for individual trials that lasted long enough to include the relevant time period. Thus, average response rates for the first two seconds of the CS could be calculated from all trials, because all trials lasted at least 2 s. For time bins beyond 2 s, however, the average response rate was calculated from the relevant proportion of trials. In this manner, response rates per time bin were corrected for opportunity of sampling. Response rates across time bins were then normalized by each mouse’s overall response rate in order to derive the proportion of responses made in each time bin. Linear slopes were then fitted to the normalized response rates. For comparisons of timing of different intervals (i.e., 10 s and 40 s), the durations were normalized by comparing responding over equivalent proportions of time.

All data were analyzed using one-way or multifactorial analysis of variance (ANOVA). The modality of the 10-s/40-s cues was included as a nuisance variable in all analyses. We have previously found that response rates are higher for auditory cues than visual cues (e.g., Sanderson, Jones, & Austen, 2016). The inclusion of this counterbalancing factor allowed assessment of the other factors independent of the variance caused by the counterbalancing factor. Consequently, in all analyses the main effect of this nuisance factor and any interactions involving the nuisance factor were ignored. Interactions were analyzed with simple main effects analysis using the pooled error term from the original ANOVA or separate ANOVAs for repeated measures with more than two levels. Where sphericity of within-subjects variables could not be assumed, a Greenhouse-Geisser correction was applied. In instances in which manipulations of the main factors of interest led to nonsignificant results, Bayesian statistics were used to evaluate the degree to which the results provided evidence for the null hypothesis. Bayesian analyses were conducted in JASP using default priors. The reported Bayes Factor compares models containing the effect of interest to equivalent models stripped of the effect, excluding higher-order interactions. The analysis was suggested by Mathôt (2017). Within JASP, this was achieved by conducting a Bayesian repeated measures ANOVA and outputting effects across matched models.

### Results and Discussion

**Figure 2 fig2:**
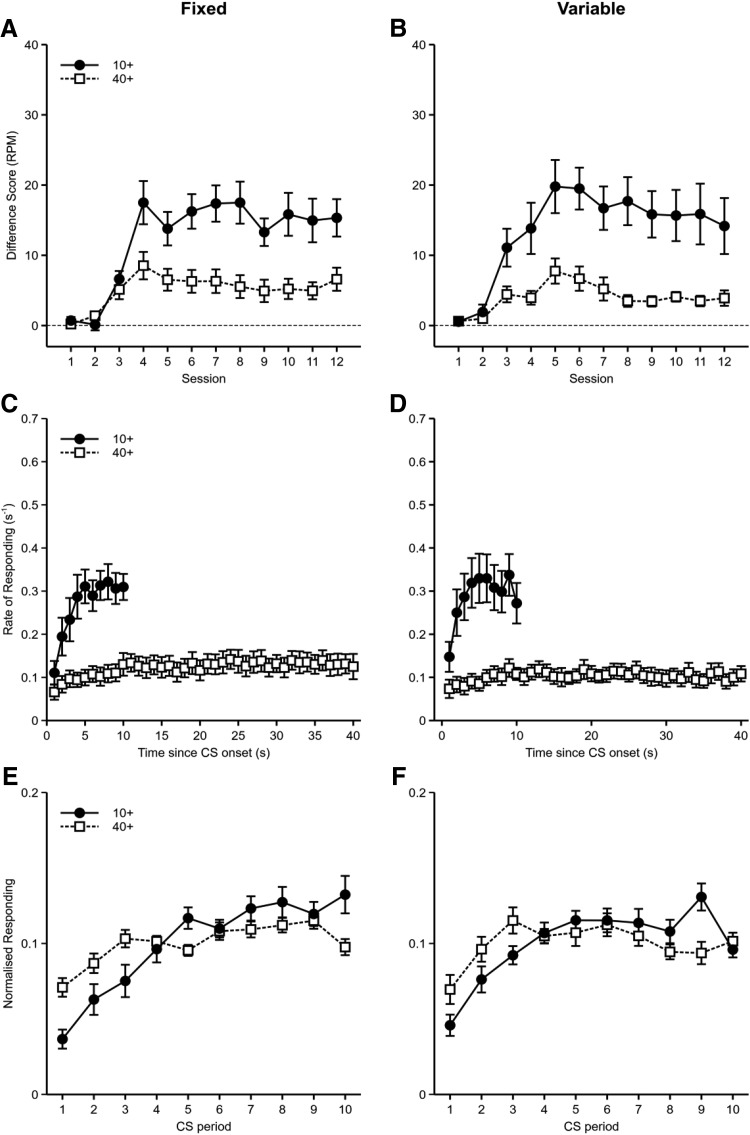
The results of Experiment 1. The results for group fixed are shown in Panels A, C, and E, and the results of group variable are shown in Panels B, D, and F. Responding across all 12 sessions of training is presented as difference scores (responses per minute [RPM]) for the 10-s cue (filled circles) and the 40-s cue (open squares) for (A) group fixed and (B) group variable. Rates of responding across the durations of the conditioned stimuli (CSs) are presented for (C) group fixed and (D) group variable. The normalized rates of responding across equivalent proportions of the 10-s and 40-s CSs are shown for (E) group fixed and (F) group variable. Error bars indicate ± *SEM*.

Responses per minute, displayed as difference scores (i.e., 10-s CS− subtracted from 10-s CS+, 40-s CS− subtracted from 40-s CS+), are shown in Figure 2A and 2B. Mice in both groups responded more to the 10-s cue than to the 40-s cue. A mixed-model ANOVA of CS Duration (10-s or 40-s) × Group (fixed or variable) × Counterbalance (10-s CS auditory or visual; nuisance factor) × Session showed significant main effects of CS duration, *F*(1, 28) = 83.1, *p* < .001, η_p_^2^ = .75, 90% confidence interval [CI] [.58, .82], and session, *F*(11, 308) = 40.5, *p* < .001, η_p_^2^ = .59, 90% CI [.52, .62], and significant Group × Session, *F*(11, 308) = 2.53, *p* = .033, η_p_^2^ = .08, 90% CI [.01, .10], and CS Duration × Session, *F*(11, 308) = 13.6, *p* < .001, η_p_^2^ = .33, 90% CI [.24, .37], interactions. The main effect of group, *F* < 1, *p* = .99, and the CS Duration × Group, *F*(1, 28) = 1.47, *p* = .24, η_p_^2^ = .05, 90% CI [.00, .22], and CS Duration × Group × Session, *F* < 1, *p* = .71, interactions were all nonsignificant. Further analysis of the significant CS Duration × Session interaction showed a significant effect of CS duration for Sessions 3–12, *F* values > 10.5, *p* values < .003. There were significant effects of session for both CS durations, *F* values > 14.3, *p* values < .001. Further analysis of the significant Group × Session interaction showed a significant effect of group for Sessions 4 and 5, *F* values > 5.9, *p* values < .022. There were significant effects of session for both groups, *F* values > 20.5, *p* values < .001.[Fig-anchor fig2]

Given that there was no significant effect of cue duration variability on responding to cues of different durations, the results fail to support the claim that response rates are determined by reinforcement rate only when responding is not timed to the occurrence of the US. To assess whether the data provide evidence for there being no effect of cue variability on sensitivity to differences in cue duration a Bayesian analysis was conducted. Although there was very strong evidence that any CS Duration × Group interaction did not interact with session, CS Duration × Group × Session *BF* = 0.01, there was moderate evidence for an interaction between CS duration and group, suggesting that the CS duration effect was larger in the variable group than the fixed group (*BF* = 5.09). Given the lack of a significant interaction in the previous ANOVA, we are cautious about making any conclusions based on this result. At the very least the Bayesian analysis did not provide strong evidence for a lack of difference in the CS duration effect between groups.

A corresponding analysis on responding during the nonreinforced cues (see Table 1 for means and *SEM*s) showed that mice responded more to the short nonreinforced cue than the long nonreinforced cue, *F*(1, 28) = 50.2, *p* < .001, η_p_^2^ = .64, 90% CI [.43, .74]. There was a significant effect of session, *F*(11, 308) = 34.7, *p* < .001, η_p_^2^ = .55, 90% CI [.48, .59], and significant Group × Session, *F*(11, 308) = 2.57, *p* = .049, η_p_^2^ = .08, 90% CI [.02, .10], and CS Duration × Session, *F*(11, 308) = 5.89, *p* < .001, η_p_^2^ = .17, 90% CI [.09, .21], interactions. The main effect of group, *F*(1, 28) = 1.46, *p* = .24, η_p_^2^ = .05, 90% CI [.00, .21], and the CS Duration × Group, *F*(1, 28) = 1.36, *p* = .25, η_p_^2^ = .05, 90% CI [.00, .21], and CS Duration × Group × Session, *F* < 1, *p* = .64, interactions were all nonsignificant. Further analysis of the significant Group × Session interaction showed a significant effect of group for Session 8 only, *F*(1, 28) = 4.78, *p* = .037. There were significant effects of session for both groups, *F* values > 16.1, *p* values < .001. Further analysis of the significant CS Duration × Session interaction showed a significant effect of CS duration, with mice responding more to the short than the long duration nonreinforced cue, on Sessions 1–7 and 10, *F* values > 4.68, *p* values < .040. There were significant effects of session for both CS durations, *F* values > 22.4, *p* values < .001. Given that mice responded more to the short duration nonreinforced cue than the long duration nonreinforced cue, this difference would have only led to underestimating the size of the CS duration effect for reinforced cues when rates of responding were converted to difference scores. Therefore, the effect of cue duration on the difference scores was not an artifact of the differences in rates of responding to the nonreinforced cues of short and long duration.[Table-anchor tbl1]

Rates of responding during the pre-CS periods were low compared to the CS periods (group fixed: *M* = 2.56 RPM ± 0.17 *SEM*; group variable: *M* = 2.43 RPM ± 0.23 *SEM*) and decreased over training (effect of session: *F*(11, 308) = 59.79, *p* < .001, η_p_^2^ = .68, 90% CI [.62, .71]). There was no significant difference between groups, *F* < 1, *p* = .59, and no significant Group × Session interaction, *F*(11, 308) = 2.18, *p* = .109, η_p_^2^ = .07, 90% CI [.01, .09]. All other main effects and interactions were also not significant (*p* values > .13).

The rates of responding across the duration of the 10 s and 40 s cues, restricted to the first 10 s and 40 s, respectively, for both groups can be seen in Figure 2C and 2D. Mice in group fixed showed an increase in responding over the duration of the cue presentations, with this increase being more pronounced for the 10 s cue than the 40 s cue. Mice in group variable also showed an increase in responding over time, but, in contrast to group fixed, responding tended to level out before the average time of reinforcement for the short and long duration cues. Timing of conditioned responding was examined by fitting linear slopes to the normalized rates of responding during comparable time periods for both groups (i.e., the 10 s of the short cue and the 40 s of the long cue for group fixed and the first 10 s of the short cue and the first 40 s of the long cue for group variable). The durations of the short and long cues were normalized by examining responding across comparable proportions of time (see Figure 2E and 2F). Thus, normalized response rates were calculated for each tenth of the short and long durations. The gradients of these normalized response rates were then calculated by fitting linear trends (fixed 10+: *M* = 0.00965, *SEM* = 0.00236; fixed 40+: *M* = 0.00314, *SEM* = 0.00092; variable 10+: *M* = 0.00565, *SEM* = 0.00146; variable 40+: *M* = 0.00104, *SEM* = 0.00136). An ANOVA of CS Duration (10-s or 40-s) × Group (fixed or variable) × Counterbalance (10-s CS auditory or visual; nuisance factor) showed that the gradients of normalized responding were steeper for the 10-s cue than for the 40-s cue, *F*(1, 28) = 21.7, *p* < .001, η_p_^2^ = .44, 90% CI [.19, .59]. Mice in the variable condition showed significantly shallower gradients than mice in the fixed condition, *F*(1, 28) = 5.19, *p* = .031, η_p_^2^ = .16, 90% CI [.01, .35]. The interaction between CS duration and group was not significant, *F* < 1, *p* = .43.

Although the distribution of responding during the CSs differed depending on whether the durations of the CSs were fixed or variable across trials, it was clear that the CS duration effect was not significantly affected by cue duration variability. The lack of difference between mice trained with variable duration CSs and those trained with fixed duration CSs is consistent with other findings in mice (Ward et al., 2012) but is in contrast to findings in rats (Jennings, Alonso, Mondragón, Franssen, & Bonardi, 2013) that showed that variable duration cues elicit weaker levels of responding compared to fixed duration cues. Also, Harris et al. (2015) found that equating reinforcement rates between continuously and partially reinforced cues led to similar levels of conditioned responding only when the cue durations were variable, but not when constant, suggesting that the opportunity to time conditioned responding affected the overall rates of responding. Our results failed to find that variable duration cues elicited weaker responding and the fact that varying the duration of cues did not affect the cue duration effect suggests differences in conditioned responding between the short and long duration cues were determined by differences in duration of cumulative exposure rather than the opportunity to time responding.

It is of note that the analysis of timing failed to show equivalent timing for short and long duration cues when the durations were normalized. It has been claimed that timing ability is scale invariant such that the variance in timing ability scales with changes in the timed duration (Gibbon, 1977). If this was the case, then the distribution of normalized responding over the short and long duration cues should be the same when responding is expressed as a function of the proportion of the timed interval. This clearly was not the case in the present experiment. This instead suggests that shorter intervals are timed better than longer intervals.

## Experiment 2a and 2b

In Experiment 1, regardless of whether responding was timed to the occurrence of reinforcement, the overall rates of responding were sensitive to the cumulative duration of the cues. Although this may suggest that delay of reinforcement is not important for determining the rate of responding, it was still true that for the group that received conditioning with variable duration cues, even though some cue presentations were very short, on average long duration cues had a longer delay of reinforcement than short duration cues.

Experiments 2a and 2b directly tested the relative contributions of delay of reinforcement and rate of reinforcement respectively. In Experiment 2a mice received conditioning with two 40-s CSs (see Figure 1C). One was reinforced after 10 s within the CS presentation and the other after 40 s, at the termination of the CS. Although the cues differed in the delay at which reinforcement occurred within the trial, both cues had the same reinforcement rate. In Experiment 2b mice were trained with a 10-s CS and a 40-s CS (see Figure 1D). Both CSs were reinforced 10-s after the onset of the cue, such that for the 40-s CS reinforcement occurred during the cue, but for the 10-s CS reinforcement occurred at the termination of the cue. Although both cues had the same delay of reinforcement, they differed in reinforcement rate.

### Method

#### Subjects and apparatus

Experiment 2a used 16 female C57BL/6J mice, 14–15 weeks old at the start of testing, with a mean free-feeding weight of 17.7*g* (range: 16.0–19.5*g*). Experiment 2b used 16 female C57BL/6J mice, 14–15 weeks old at the start of testing, with a mean free-feeding weight of 18.7*g* (range: 16.0–21.4*g*). Mice for both experiments had previously been used in an unrelated experiment involving consumption of flavoured sucrose solutions, conducted in a different room in operant boxes that were distinct from those used in the current experiment. All other details were the same as Experiment 1.

#### Procedure

In Experiment 2a, mice received 12 sessions of training with four 40-s cues (two auditory and two visual). One of the cues was reinforced by the presentation of a sucrose pellet 10 s into the presentation of the cue (40/10+, see Figure 1C). Another cue was reinforced by the presentation of a sucrose pellet after 40 s, at the termination of the cue (40/40+, see Figure 1C). The remaining cues were nonreinforced (CS−). For half of the mice the modality of the cue reinforced after 10 s was auditory (noise, clicker) and the modality of the cue reinforced after 40 s was visual (house light, flashing LEDs with alternating 0.5 s illumination of the left and right LEDs). The opposite was true for the remaining mice. Within each of these subgroups the identity of the reinforced and nonreinforced stimuli was fully counterbalanced. In Experiment 2b mice received 12 sessions of training with two 10-s duration cues and two 40-s cues. One cue of each duration was reinforced. The 10-s cue was reinforced by the presentation of a sucrose pellet after 10 s, at the termination of the cue (10/10+, see Figure 1D). The 40-s cue was reinforced by the presentation of a sucrose pellet 10 s into the presentation of the cue (40/10+, see Figure 1D). The remaining short and long cues were nonreinforced. For half of the mice, the short cues were auditory (noise, clicker) and the long cues were visual (house light, flashing LEDs with alternating 0.5-s illumination of the left and right LEDs). The opposite was true for the remaining mice. Within each of these subgroups the identity of the reinforced and nonreinforced stimuli was fully counterbalanced. For both experiments mice received six presentations of each cue per session, with a fixed interval of 120 s between the offset of one cue and the onset of the next. The order of trials was random with the constraint that there was an equal number of each trial type every eight trials. For each session all mice received the stimuli presented in the same order (e.g., 1st trial = noise, 2nd trial = house light, 3rd trial = clicker etc.). Because the identity of the stimuli used in the different conditions was counterbalanced across mice, this resulted in the order of the different conditions across trials also being counterbalanced across mice.

#### Data analysis

The frequency of head entries into the food magazine was recorded per-second during the CS exposure prior to the presentation of reinforcement. Therefore, in the condition in which the 40-s cue was reinforced 10 s after the CS onset, rates of responding are reported for the initial 10 s of the CS and not the subsequent 30 s after reinforcement. Rates of responding during reinforced cues were converted to difference scores by subtracting the rate of responding during the equivalent period of the nonreinforced cue of the same modality. Responding was also recorded for a 10-s pre-CS period. All other details were the same as Experiment 1.

### Results and Discussion

#### Experiment 2a

**Figure 3 fig3:**
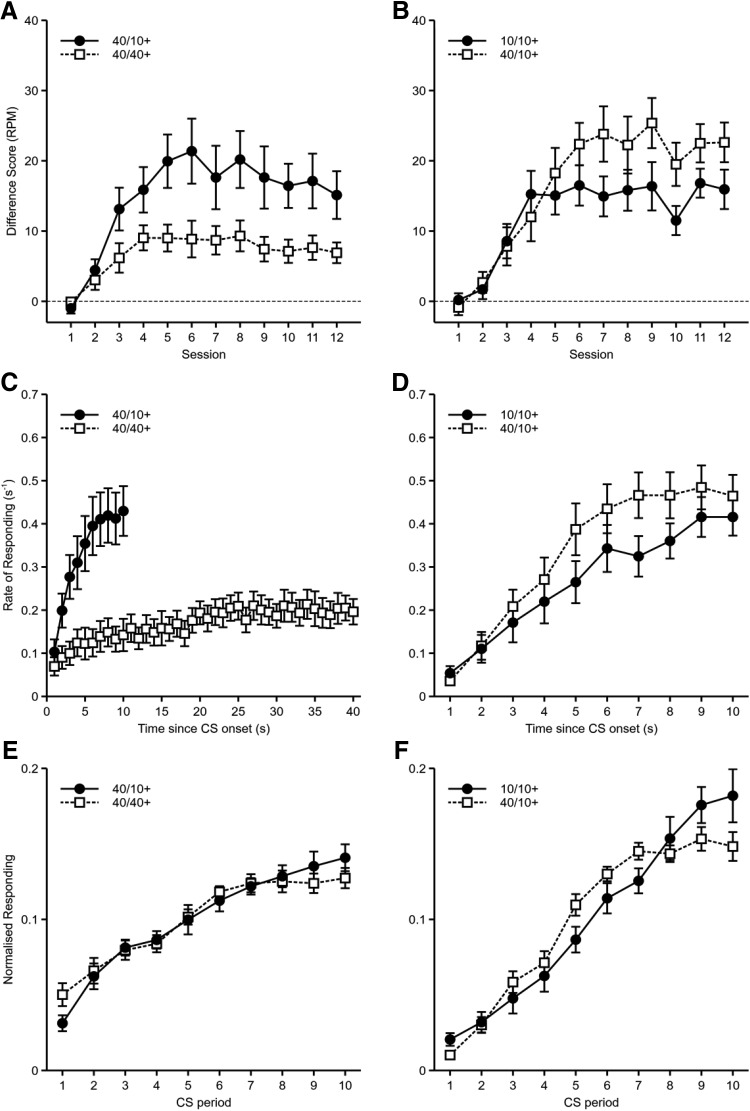
The results of Experiments 2a and 2b. The results of Experiment 2a are shown in Panels A, C, and E. The results of Experiment 2b are shown in Panels B, D, and F. Responding across all 12 sessions of training is presented as difference scores (responses per minute [RPM]) for the 40/10+ cue in Experiment 2a and the 10/10+ cue in Experiment 2b (filled circles) and for the 40/40+ cue in Experiment 2a and the 40/10+ cue in Experiment 2b (open squares) for (A) Experiment 2a and (B) Experiment 2b. Rates of responding across the durations of the conditioned stimuli (CSs) prior to reinforcement are shown for (C) Experiment 2a and (D) Experiment 2b. The normalized rates of responding across equivalent proportions of the cue duration are presented for (E) Experiment 2a and (F) Experiment 2b. Error bars indicate ± *SEM*.

Responses per minute, displayed as difference scores (rate of responding during the reinforced cues, prior to reinforcement, minus the rate of responding during the equivalent periods for the nonreinforced cues of the same modality as the reinforced cue) are shown in Figure 3A. Mice responded more in the 40/10+ condition than in the 40/40+ condition. An ANOVA of Delay (40/10+ vs. 40/40+) × Counterbalance (40/10+ CS auditory or visual; nuisance factor) × Session showed significant main effects of delay, *F*(1, 14) = 20.9, *p* < .001, η_p_^2^ = .60, 90% CI [.26, .74], and session, *F*(11, 154) = 17.0, *p* < .001, η_p_^2^ = .55, 90% CI [.43, .59], and a significant Delay × Session interaction, *F*(11, 154) = 3.43, *p* = .008, η_p_^2^ = .20, 90% CI [.06, .23]. Further analysis of this interaction showed that responding in the 40/10+ condition was higher than responding in the 40/40+ condition on Sessions 3–12, *F* values > 5.2, *p* values < .04.[Fig-anchor fig3]

A corresponding analysis of responding during the nonreinforced cues (see Table 2 for means and *SEM*s) showed that mice responded more to the nonreinforced cue that was of the same modality as the cue that was reinforced after 10 s compared to the nonreinforced cue that was the same modality as the cue reinforced after 40 s, *F*(1, 14) = 11.2, *p* = .005, η_p_^2^ = .44, 90% CI [.10, .63]. Response rates tended to decrease over sessions, *F*(11, 154) = 14.2, *p* < .001, η_p_^2^ = .50, 90% CI [.38, .55]. There was no significant Delay × Session interaction, *F*(11, 154) = 1.70, *p* = .15, η_p_^2^ = .11, 90% CI [.00, .13]. Similar to Experiment 1, the difference between the nonreinforced cues would have only led to underestimating the size of the effect of delay for reinforced cues when rates of responding were converted to difference scores. Therefore, the effect of delay on the difference scores was not an artifact of the differences in rates of responding to the nonreinforced cues.[Table-anchor tbl2]

The rates of responding during the pre-CS periods were low (*M* = 1.77 RPM ± 0.12 *SEM*) and decreased over training, *F*(11, 154) = 16.39 *p* < .001, η_p_^2^ = .54, 90% CI [.42, .58]. There were no other significant main effects or interactions (smallest *p* value = .071).

The rate of responding during each second of the reinforced cues is shown in Figure 3C. For both reinforced cues the rate of responding increased over the course of the cue. As for Experiment 1, timing was analyzed by normalizing response rates across equivalent proportions of the 10-s and 40-s delays (see Figure 3E). The gradients of these normalized response rates were then calculated by fitting linear trends (40/10+: *M* = 0.01123, *SEM* = 0.00079; 40/40+: *M* = 0.00887, *SEM* = 0.00172). There was no significant difference in the gradients between the two conditions, *F*(1, 14) = 1.93, *p* = .19, η_p_^2^ = .12, 90% CI [.00, .37]. This is in contrast to Experiment 1, in which we found a significant difference in the distribution of responses for the short and long delays of reinforcement. It is not clear why the results of the two experiments differ. One potential difference in Experiment 2a was the degree of temporal contiguity between the CS and US for each delay. This is discussed further below.

#### Experiment 2b

Responses per minute, displayed as difference scores (i.e., 10/10 CS− subtracted from 10/10 CS+, 40/10 CS− subtracted from 40/10 CS+), are shown in Figure 3B. Mice responded more in the 40/10+ condition than in the 10/10+ condition. An ANOVA of Reinforcement Rate (10/10 or 40/10) × Counterbalance (10/10+ CS auditory or visual; nuisance factor) × Session showed significant main effects of reinforcement rate, *F*(1, 14) = 18.7, *p* = .001, η_p_^2^ = .57, 90% CI [.22, .74], and session, *F*(11, 154) = 24.2, *p* < .001, η_p_^2^ = .63, 90% CI [.53, .67], and a significant Reinforcement Rate × Session interaction, *F*(11, 154) = 3.18, *p* = .001, η_p_^2^ = .19, 90% CI [.05, .22]. Further analysis of this interaction showed that responding in the 40/10+ condition was higher than in the 10/10+ condition on Sessions 6, 7, 9, 10, 11, and 12, *F* values > 7.0, *p* values < .02.

A corresponding analysis of responding during the nonreinforced cues (see Table 2 for means and *SEM*s) showed a significant main effect of session, *F*(11, 154) = 10.4, *p* < .001, η_p_^2^ = .43, 90% CI [.29, .47], but no significant main effect of reinforcement rate, *F* < 1, *p* = .60, and no significant Reinforcement Rate × Session interaction, *F* < 1, *p* = .84.

The rates of responding during the pre-CS periods were low (*M* = 3.42 RPM ± 0.31 *SEM*) and decreased over training, *F*(11, 154) = 11.12 *p* < .001, η_p_^2^ = .44, 90% CI [.31, .49]. There were no other significant main effects or interactions (smallest *p* value = .053).

The rate of responding during each second of the CS+ is shown in Figure 3D. For both reinforced cues the rate of responding increased over the course of the cue. There was no significant difference in the gradients of normalized responding between the two conditions (10/10+: *M* = 0.01942, *SEM* = 0.00241; 40/10+: *M* = 0.01680, *SEM* = 0.00144), *F*(1, 14) = 3.05, *p* = .10, η_p_^2^ = .18, 90% CI [.00, .43] (Figure 3F).

In Experiment 2a, it was found that a shorter delay of reinforcement within a CS presentation led to greater responding compared to a longer delay of reinforcement, even though both CSs were matched for their overall rate of reinforcement. In Experiment 2b, even though the CSs were matched for delay of reinforcement, it was found that the CS that had a lower reinforcement rate elicited greater responding than the CS with the higher reinforcement rate. Although the results of these two experiments contradict previous results, suggesting that increases in reinforcement rate do not necessarily lead to increases in response rate, these results can, instead, be explained in terms of differences in temporal contiguity. In Experiment 2a, when the CS with the long delay of reinforcement was reinforced, the pellet was presented at the termination of the CS and mice would have consumed the pellet some moments later. For the short delay of reinforcement CS, however, the pellet was presented during the CS and mice would have likely consumed the pellet during the continued presentation of the CS. Therefore, the greater temporal contiguity between the CS and reinforcement may have led to the greater rate of responding for the short delay of reinforcement CS compared to the long delay of reinforcement CS.

Temporal contiguity between the CS and reinforcement may also explain the performance of mice in Experiment 2b. For the 40-s CS that was reinforced after 10 s, consumption of the pellet would have likely been contiguous with the presentation of the CS. For the 10-s CS, however, mice would have consumed the pellet some moments after the termination of the CS. Therefore, despite the 40-s CS having a lower reinforcement rate than the 10-s CS, the degree of temporal contiguity between the cue and reinforcement would have been higher.

Other experiments that have compared conditioning with a CS that is extended past the presentation of the US with a CS that has terminated at the onset of the US have found mixed results that may reflect differences in the nature of the conditioned response that was measured (Wagner & Brandon, 1989). For example, extending the duration of the CS after the onset of shock reduces conditioned suppression in rats (Ayres & Albert, 1990; Ayres, Albert, & Bombace, 1987), but similar manipulations, using conditioning of the nictitating membrane response in rabbits, increases conditioning (Kehoe, 2000). Although the results of Experiment 2a and 2b are consistent with the proposal that the offset of a CS engages inhibitory processes (Klopf, 1988), the fact that it is not possible to have high temporal control over the consumption of the food pellet makes it likely that the superior acquisition of responding with the CS that was extended past the US reflects increased temporal contiguity.

Given the confound in the degree of temporal contiguity between the CS and US, it is clear that Experiments 2a and 2b did not provide an unambiguous test of delay of reinforcement and rate of reinforcement. The results of these two experiments do suggest, however, that temporal contiguity has a greater effect on responding than delay and rate of reinforcement. Thus, demonstrations of the role of reinforcement rate, and any potential role of delay of reinforcement, may only be revealed under conditions in which the temporal contiguity between events is equal.

## Experiment 3

The purpose of Experiment 3 was to test the role of delay of reinforcement and rate of reinforcement under conditions in which differences in the degree of temporal contiguity between the CS and reinforcement were avoided, or at the very least substantially reduced. Four groups of mice were used. Two groups of mice were trained with a 40-s and a 70-s CS. For half of these mice, reinforcement occurred 10 s after the start of both CSs (40/10+ and 70/10+, see Figure 1E). For the other half, the 40-s CS was reinforced 10 s after the start of the CS (40/10+), and the 70-s CS was reinforced 40 s after the start of the CS (70/40+). For both of these groups reinforcement was presented during the CS and there was a substantial period of time (at least 30 s) left within the CS presentation in which mice would have likely consumed the pellet. Therefore, the degree of temporal contiguity between reinforcement and the CS would have been similar between the CSs. Certainly, temporal contiguity was matched for the group in which the 40-s CS was reinforced after 10 s and the 70-s CS was reinforced after 40 s, because both CSs were presented for 30 s after the presentation of reinforcement. For the first group of mice delay of reinforcement was matched, but the rate of reinforcement was lower for the 70-s CS than for the 40-s CS. In contrast, delay of reinforcement and rate of reinforcement was confounded in the second group such that the 70-s CS had a longer delay of reinforcement and lower reinforcement rate than the 40-s CS. If the CS duration effect was weaker for the group in which delay of reinforcement was matched compared to the group in which it was not, then it would suggest that delay of reinforcement plays a role, independent of reinforcement rate, in determining the cue duration effect.

The two other groups of mice received similar training to the first two groups, with the exception that the long duration CS was 160 s rather than 70 s. Therefore, one group was trained with a 40-s and 160-s CS and both CSs were reinforced after 10 s (40/10+ and 160/10+, see Figure 1E). For the last group the 40-s CS was reinforced after 10 s (40/10+) and the 160-s CS was reinforced after 40 s (160/40+). For these groups there was a fourfold difference in reinforcement rate between the short and long duration CSs, matching the proportional difference in delay of reinforcement for the group that received the reinforcement after 40 s during the 160-s long-duration CS.

### Method

#### Subjects and apparatus

Sixty-four experimentally naïve female C57BL/6J mice were used. They were 10–16 weeks old at the start of testing, with a mean free-feeding weight of 19.7*g* (range: 16.9 – 23.0*g*). All other details were the same as Experiment 1.

#### Procedure

Mice received 12 sessions of training, one per day. All mice received training with two 40-s cues, one of which was reinforced after 10 s, and the other was nonreinforced. Mice also received training with a longer duration cue that was reinforced after either the same or longer delay as the reinforced 40-s cue. Half of the mice received additional training with two 70-s cues. The remaining mice received training with two additional 160-s cues. For half of the mice trained with either 70-s or 160-s long cues, one long (70 s/160 s) cue was reinforced by presentation of a sucrose pellet after 10 s. For the remaining mice the long cue (70 s/160 s) was reinforced after 40 s. Therefore, mice were trained in one of four groups, in two of which the long cue had the same time of reinforcement as the short cue (group 70/10+ and group 160/10+), and for the other two the long cue had a different time of reinforcement to the short cue (group 70/40+ and group 160/40+). The remaining short and long duration cues were nonreinforced. Within each group, for half of the mice the short (40 s) cues were auditory (noise, clicker) and the long (70 s/160 s) cues were visual (house light, flashing LEDs with alternating 0.5 s illumination of the left and right LEDs). The opposite was true for the remaining mice. Within each of these subgroups the identity of the reinforced and nonreinforced stimuli was fully counterbalanced. Each of the four cues was presented six times per session with a fixed interval of 120 s between the offset of one cue and the onset of the next. Trials were presented in a random order with the constraint that an equal number of each cue was presented every block of eight trials. For each session all mice received the stimuli presented in the same order (e.g., 1st trial = noise, 2nd trial = house light, 3rd trial = clicker etc.). Because the identity of short- and long-duration cues and the identity of reinforced and nonreinforced cues were counterbalanced across mice, this resulted in the order of these factors also being counterbalanced across mice.

#### Data analysis

The frequency of head entries into the food magazine was recorded per-second during the CS exposure prior to the presentation of reinforcement. Rates of responding during reinforced cues were converted to difference scores by subtracting the rate of responding during the equivalent period of the nonreinforced cue of the same modality. Responding was also recorded for a 10-s pre-CS period. All other details were the same as Experiment 1.

### Results and Discussion

**Figure 4 fig4:**
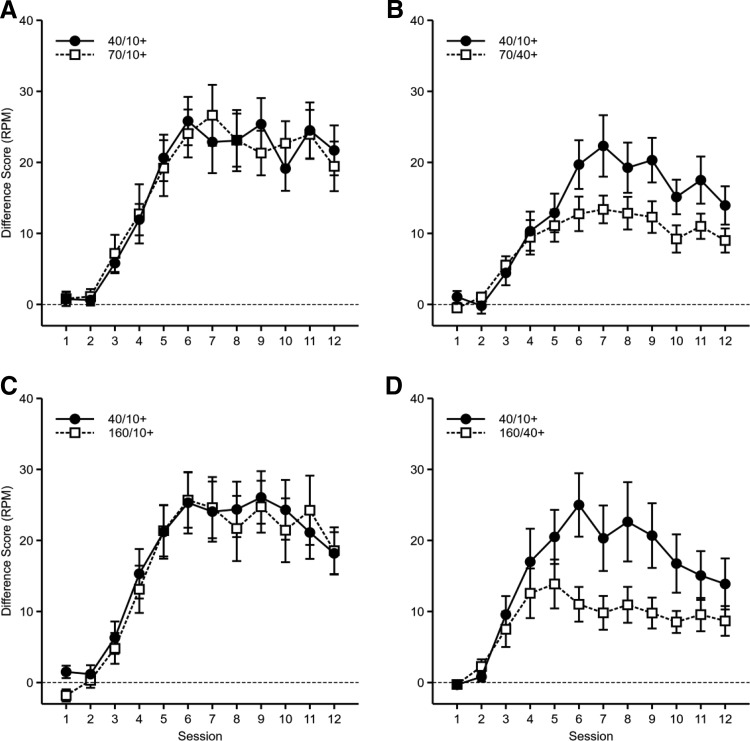
The rates of responding for Experiment 3. Responding across all 12 sessions of training is shown as difference scores (responses per minute [RPM]) for the short duration cue (filled circles) and the long duration cue (open squares) for the four groups of Experiment 3. (A) Group 70/10+; (B) Group 70/40+; (C) Group 160/10+; (D) Group 160/40+. Error bars indicate ± *SEM*.

Responses per minute, displayed as difference scores, are shown in Figure 4A–4D. Mice responded similarly to all cues that were reinforced after 10 s, but responded less to those cues that were reinforced after 40 s. This was true regardless of whether the long duration cue was 70 s or 160 s. An ANOVA of Cue Duration (short [40 s] vs. long [70 s/160 s]) × Delay of Reinforcement of Long Cue (10 s or 40 s) × Duration of Long Cue (70 s or 160 s) × Counterbalance (40/10+ CS auditory or visual; nuisance factor) × Session was conducted. There were significant main effects of cue duration, *F*(1, 56) = 9.76, *p* = .003, η_p_^2^ = .15, 90% CI [.03, .29], session, *F*(11, 616) = 96.2, *p* < .001, η_p_^2^ = .63, 90% CI [.59, .66], and delay of reinforcement of long cue, *F*(1, 56) = 18.0, *p* < .001, η_p_^2^ = .24, 90% CI [.09, .38]. There was a significant Cue Duration × Delay of Reinforcement of Long Cue interaction, *F*(1, 56) = 7.11, *p* = .010, η_p_^2^ = .11, 90% CI [.02, .25]. Each of these factors also interacted with session: Session × Delay of Reinforcement of Long Cue, *F*(11, 616) = 7.17, *p* < .001, η_p_^2^ = .11, 90% CI [.06, .14]; Cue Duration × Session, *F*(11, 616) = 2.98, *p* = .013, η_p_^2^ = .05, 90% CI [.01, .06]. Furthermore, there was a significant three-way interaction between cue duration, delay of reinforcement of long cue, and session, *F*(11, 616) = 3.17, *p* = .009, η_p_^2^ = .05, 95% CI [.01, .07]. All other main effects and interactions were nonsignificant, *F* values < 1.3, *p* values > .3. Further analysis of the Cue Duration × Delay of Reinforcement of Long Cue interaction showed that when the long cue was reinforced after 10 s, responding was similar for both the short and long duration cues, *F* < 1, *p* = .75. When the long cue was reinforced after 40 s, responding to the short cue was greater than to the long cue, *F*(1, 56) = 16.8, *p* < .001. Responding to the long cue was significantly higher when reinforced after 10 s rather than 40 s, *F*(1, 56) = 25.5, *p* < .001, but this comparison did not reach significance for responding to the short cue, *F*(1, 56) = 3.60, *p* = .063.[Fig-anchor fig4]

The results demonstrate that when cues were matched for delay of reinforcement there was no significant effect of reinforcement rate. To assess whether the data provided evidence for there being no effect of reinforcement rate when delay of reinforcement was controlled a Bayesian analysis was conducted. There was strong evidence for a lack of effect of cue duration (*BF* = 0.089) and a lack of a Cue Duration × Session interaction (*BF* = 0.001) for those animals for which the long cue was reinforced after 10 s.

A corresponding analysis of responding during the nonreinforced cues (see Table 3 for means and *SEM*s) was conducted. Mice responded more to the short duration cue than the long duration cue and this effect was greater for groups in which the long duration cue was reinforced after 40 s compared to those for which reinforcement occurred after 10 s. There was a significant effect of cue duration, *F*(1, 56) = 22.0, *p* < .001, η_p_^2^ = .28, 90% CI [.12, .42], and session, *F*(11, 616) = 24.1, *p* < .001, η_p_^2^ = .30, 90% CI [.24, .34]. There were also significant Cue Duration × Delay of Reinforcement of Long Cue, *F*(1, 56) = 10.2, *p* = .002, η_p_^2^ = .15, 90% CI [.04, .29], and Cue Duration × Session, *F*(11, 616) = 6.26, *p* < .001, η_p_^2^ = .10, 90% CI [.05, .12], interactions. All other main effects and interactions were nonsignificant, *F* values < 1.6, *p* values > .14. Further analysis of the Cue Duration × Delay of Reinforcement of Long Cue interaction showed a significant effect of delay of reinforcement of long cue for the long duration cue, *F*(1, 56) = 16.4, *p* < .001, but not for the short duration cue, *F*(1, 56) = 1.06, *p* = .31. In addition, there was an effect of cue duration when the delay of reinforcement for the short and long duration cues differed, *F*(1, 56) = 31.1, *p* < .001, but not when matched, *F*(1, 56) = 1.11, *p* = .30. Given that the difference between nonreinforced cues followed the same pattern as for the difference scores, it is unlikely that the effects found with the difference scores were an artifact of differences in responding to the nonreinforced cues.[Table-anchor tbl3]

The rates of responding during the pre-CS periods were low (group 70/10+: *M* = 2.55 RPM ± 0.27 *SEM*; group 70/40+: *M* = 2.40 RPM ± 0.23 *SEM*; group 160/10+: *M* = 2.56 RPM ± 0.21 *SEM*; group 160/40+: *M* = 2.78 RPM ± 0.21 *SEM*) and reduced over training, *F*(11, 616) = 42.64, *p* < .001, η_p_^2^ = .43, 90% CI [.38, .47]. The between-subjects manipulations of the duration of long cue and the delay of reinforcement of the long cue were not significant and did not interact with other factors (*p* values > .11). Responding was higher prior to short duration cues compared to long duration cues—short [40 s]: *M* = 2.67 RPM ± 0.12 *SEM*; long [70/160 s]: *M* = 2.47 RPM ± 0.11 *SEM*, *F*(1, 56) = 7.50, *p* = .008, η_p_^2^ = .12, 90% CI [.02, .25]—and rates of responding prior to reinforced and nonreinforced cues interacted with session, *F*(11, 616) = 2.05, *p* = .040, η_p_^2^ = .04, 90% CI [.003, .04]. None of these differences in baseline responding can account for the patterns of responding to the reinforced cues as measured by the difference scores. In addition, given that the trial order of cues was counterbalanced in terms of whether they were of a short or long duration or were reinforced or nonreinforced, these differences likely reflect chance variation in response rates. There were no other significant effects or interactions, *p* values > .11.

**Figure 5 fig5:**
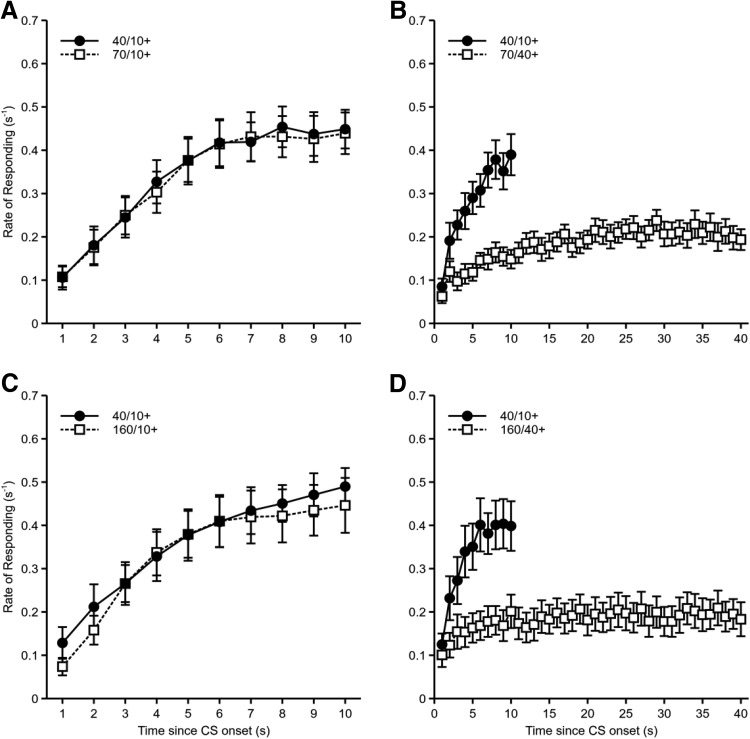
The rate of responding across the conditioned stimulus (CS) duration prior to reinforcement for Experiment 3. The short duration cue is shown by the filled circles and the long duration cue is shown by the open squares. (A) Group 70/10+; (B) Group 70/40+; (C) Group 160/10+; (D) Group 160/40+. Error bars indicate ± *SEM*.

**Figure 6 fig6:**
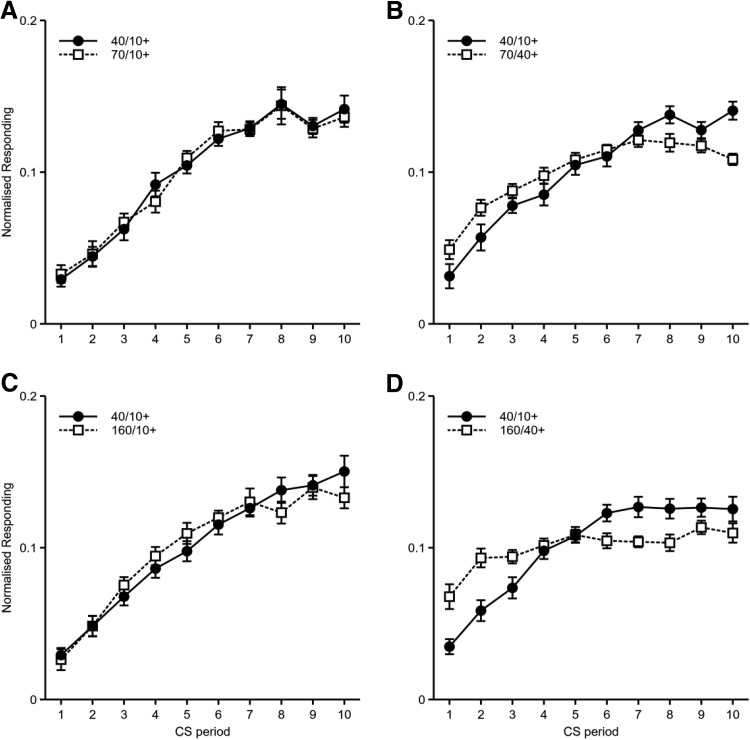
The normalized rates of responding across equivalent proportions of the cue duration for Experiment 3. (A) Group 70/10+; (B) Group 70/40+; (C) Group 160/10+; (D) Group 160/40+. Error bars indicate ± *SEM*.

The rates of responding during each second of the short and long duration CSs are shown in Figure 5. The rates of responding were similar for those cues reinforced after 10 s, but rate of responding was lower for the cues reinforced after 40 s. Analysis of the gradients of normalized responding (group 70/10+: 40/10+: *M* = 0.01305, *SEM* = 0.00149; 70/10+: *M* = 0.01244, *SEM* = 0.00146; group 70/40+: 40/10+: *M* = 0.01157, *SEM* = 0.00120; 70/40+: *M* = 0.00640, *SEM* = 0.00113; group 160/10+: 40/10+: *M* = 0.01349, *SEM* = 0.00156; 160/10+: *M* = 0.01185, *SEM* = 0.00096; group 160/40+: 40/10+: *M* = 0.01002, *SEM* = 0.00129; 160/40+: *M* = 0.00344, *SEM* = 0.00111; see Figure 6) across equivalent proportions of the delay to reinforcement showed significant main effects of cue duration, *F*(1, 56) = 26.5, *p* < .001, η_p_^2^ = .32, 90% CI [.16, .45], and delay of reinforcement of long cue, *F*(1, 56) = 39.9, *p* < .001, η_p_^2^ = .42, 90% CI [.25, .54], and a significant interaction between these factors, *F*(1, 56) = 12.2, *p* = .001, η_p_^2^ = .18, 90% CI [.05, .32] (see Figure 6). All other main effects and interactions were not significant, *F* values < 2.02, *p* values > .16. Further analysis of the Cue Duration × Delay of Reinforcement of Long Cue interaction showed that for animals for which the long cue was reinforced after 40 s, the responding gradient was steeper for the short cue than for the long cue, *F*(1, 56) = 37.4, *p* < .001, but this was not the case when the delay of reinforcement was matched between short and long duration cues, *F*(1, 56) = 1.37, *p* = .25. Gradients for the long cue were significantly shallower when it was reinforced after 40 s compared to when it was reinforced after 10 s, *F*(1, 56) = 48.66, *p* < .001. The gradients for the short duration cue were also affected by the delay of reinforcement for the long duration cue, with gradients being shallower when the delay of reinforcement for the long duration cue was 40 s compared to 10 s, *F*(1, 56) = 5.93, *p* = .018 (see Figure 6). The difference between short and long delays of reinforcement on the distribution of responding replicates the effect found in Experiment 1, suggesting that timing does not scale with duration. The fact that mice trained with a 40-s delay of reinforcement showed worse timing of the 10-s cue compared to mice that only ever received reinforcement after a 10-s delay suggests that there was some generalization or interference between the 10-s and 40-s intervals that affected timing of conditioned responses to the short duration cue.[Fig-anchor fig5][Fig-anchor fig6]

When the time of reinforcement was matched across CSs that differed in reinforcement rate, the rates of responding were similar. In contrast, a CS that was reinforced after 40 s elicited weaker responding than a CS that was reinforced after 10 s. By comparing across groups, it was clear that there was an effect of delay of reinforcement even when cues were matched for reinforcement rate. These results suggest that the time that reinforcement occurs within a cue is more important than the rate at which reinforcement occurs across the CS for determining response rates. In addition, it was clear that reinforcement rates failed to affect timing of conditioned responding.

Given that the results of Experiment 2 suggested that presenting reinforcement during the presentation of a CS, prior to termination of the cue, aids conditioning by increasing temporal contiguity, it is possible that reinforcing a cue earlier in its presentation increased temporal contiguity in Experiment 3. Thus, the long duration CSs may have had increased temporal contiguity with reinforcement when reinforced after 10 s compared to when reinforced after 40 s. This, however, seems unlikely. Sanderson, Cuell, and Bannerman (2014) have shown in mice, using a similar conditioning procedure, that when a presentation of a pellet preceded a CS by 10 s there was no excitatory conditioning (see also Delamater, Sosa, & LoLordo, 2003). In the present experiment, in all conditions, the long-duration cue was presented for at least 30 s after the presentation of the pellet. Furthermore, even if the reinforcing effects persisted for more than 30 s, it is unlikely that differences in temporal contiguity could account for differences in the rates of responding to the 160-s cue when reinforced after 10 or 40 s (i.e., it is unlikely that an extra 150 s of exposure after the US will lead to greater temporal contiguity that an extra 120 s). There was also no evidence that longer cues elicited greater responding than shorter duration cues when the delay of reinforcement was matched. Therefore, the results suggest that, in the absence of differences in temporal contiguity, delay of reinforcement has a greater effect on conditioned responding than rate of reinforcement.

## Experiment 4

In Experiment 3 conditioned responding was unaffected by differences in cue duration (i.e., 40 s, 70 s and 160 s) if the cues were reinforced after 10 s from their onset. This suggests that differences in the duration of nonreinforcement after reinforcement, within the trial, failed to affect performance. Therefore, the subsequent 150 s of nonreinforced exposure after reinforcement in the 160 s cue did not reduce responding on subsequent trials compared to a cue that was four times shorter. There may be a number of possible explanations for why this nonreinforced exposure failed to extinguish responding. One simple possibility is that the nonreinforced exposure was not processed sufficiently for extinction to occur, perhaps as a consequence of the recent reinforcement. The purpose of Experiment 4 was to test whether mice are able to learn about events that occur after a US presentation, but that occur within the same trial.

In Experiment 4 mice received conditioning with two cues that were 80 s in duration. Both cues were reinforced 10 s after the onset of the cue. One cue was reinforced again 30 s later, 40 s after the onset of the cue (10+/40+), but the other cue was not (10+/40−). Mice also received training with a third cue that was not reinforced and served as a control cue to determine baseline levels of responding.

### Method

#### Subjects and apparatus

Twenty-four experimentally naïve female C57BL/6J mice were used. They were 10–11 weeks old at the start of testing, with a mean free-feeding weight of 18.8*g* (range: 16.9–21.0*g*). All other details were the same as Experiment 1, with the addition of a pure tone generator (ENV-323AM) that produced a 2,900 Hz tone at 75 dB.

#### Procedure

Mice received 10 sessions of training with three auditory cues (tone, noise, and clicker), each with a duration of 80 s. During training, one cue (10+/40−) was reinforced with a sucrose pellet 10 s after cue onset. Another cue (10+/40+) was similarly reinforced after 10 s, but also reinforced again 40 s after onset. A third cue (CS−) was nonreinforced. During each session, there were eight presentations of each cue, separated by a fixed interval of 120 s. Cues were presented in a random order with the constraint that there were four of each type every 12 trials. The allocation of the tone, noise, and clicker to the three conditions (10+/40−, 10+/40+, and CS−) was counterbalanced across mice using equal numbers of the six possible combinations of cues. For each session all mice received the stimuli presented in the same order (e.g., 1st trial = noise, 2nd trial = tone, 3rd trial = clicker etc.). Because the identity of the stimuli assigned to the different conditions was counterbalanced across mice, this resulted in the order of these conditions across trials also being counterbalanced across mice.

#### Data analysis

Responding was analyzed during seconds 1–10 and 31–40 for each cue, corresponding to the 10 s prior to the delivery of the two reinforcements for the 10+/40+ cue and allowing comparable control periods for the other cues. Rates of responding during the reinforced cues were converted to difference scores by subtracting the rate of responding during the equivalent period of the nonreinforced cue. Responding was also recorded for the 10-s pre-CS period. All other details were the same as Experiment 1.

### Results and Discussion

**Figure 7 fig7:**
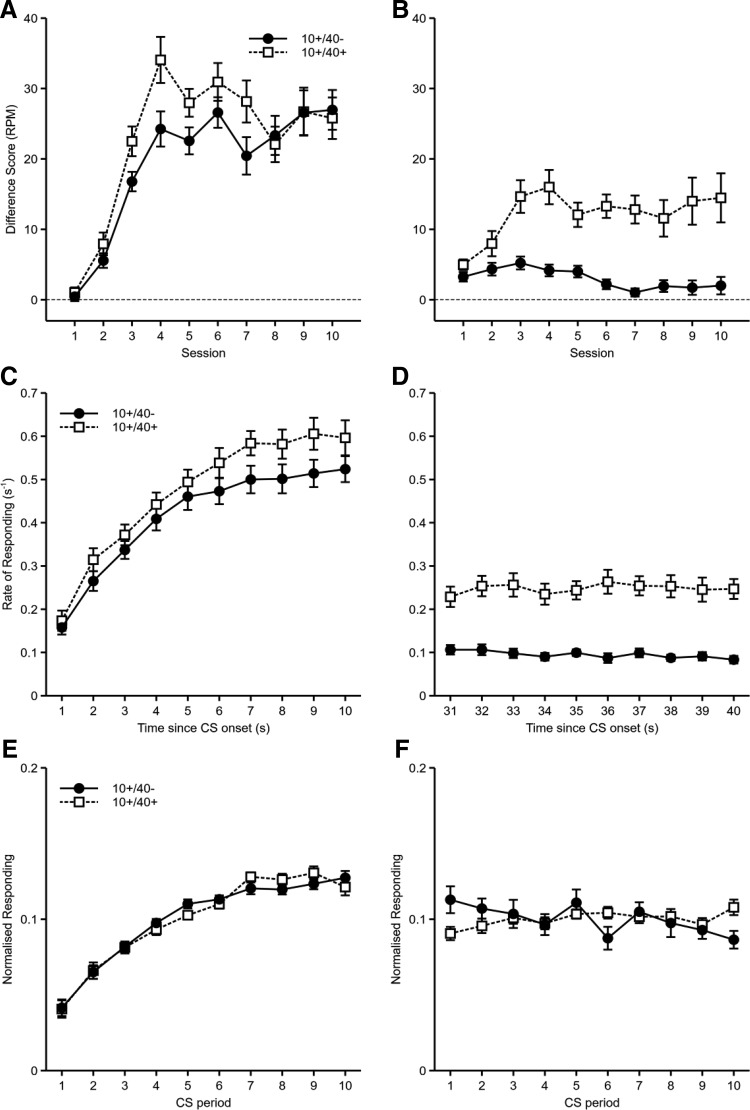
The results of Experiment 4. The results for the first period (1–10 s) are shown in Panels A, C, and E. The results for the second period (31–40 s) are shown in Panels B, D, and F. Responding across all 10 sessions of training is presented as difference scores (responses per minute [RPM]) for the 10+/40− cue (filled circles) and the 10+/40+ cue (open squares) for (A) conditioned stimulus (CS) period 1–10 s and (B) CS period 31–40 s. Rates of responding across the durations of the CSs during the first period (1–10 s) are shown in Panel C and the second period (31–40 s) are shown in Panel D. The normalized rates of responding across equivalent proportions of the cue duration are shown for the first period (1–10 s) in Panel E and the second period (31–40 s) in Panel F. Error bars indicate ± *SEM*.

The rates of responding during the first 10 s and the period from the 31st to the 40th second are shown in Figure 7A–7B. Mice responded more to cue 10+/40+ than to cue 10+/40− during both periods, although by the end of training this difference was not present in the first period. A Cue (10+/40− vs. 10+/40+) × Period (1–10 s vs. 31–40 s) × Session ANOVA revealed a significant three-way interaction between factors, *F*(9, 207) = 4.53, *p* = .001, η_p_^2^ = .16, 90% CI [.06, .21]. All other main effects and interactions were also significant (*p* values < .005). The three-way interaction was analyzed by conducting separate ANOVAs for the first (1–10 s) and second (31–40 s) periods. For the first period (1–10 s) there was a significant Cue × Session interaction, *F*(9, 207) = 4.54, *p* < .001. Simple main effects analysis of the interaction revealed that there were significant effects of cue on Sessions 3–5 and 7, smallest *F*(1, 23) = 4.83 (*p* = .038), but not on the other sessions (*p* values > .08). For the second period (31–40 s) there was also a significant Cue × Session interaction, *F*(9, 207) = 3.95, *p* = .015. Simple main effects analysis revealed a significant effect of cue on Sessions 1 and 3–10, smallest *F*(1, 23) = 4.43 (*p* = .047), but not Session 2 (*p* = .071).[Fig-anchor fig7]

An analysis of responding during the two periods for the CS− was also conducted (see Table 4 for means and *SEM*s). Mice responded more during the first period than the second, *F*(1, 23) = 36.89, *p* < .001, η_p_^2^ = .62, 90% CI [.37, .73]. Responding also decreased over sessions, *F*(9, 207) = 17.32, *p* < .001, η_p_^2^ = .43, 90% CI [.32, .48]. There was also a significant Period × Session interaction, *F*(9, 207) = 5.40, *p* < .001, η_p_^2^ = .19, 90% CI [.09, .24]. Simple main effects analysis of the interaction revealed that there was a significant effect of period on Sessions 2–9, smallest *F*(1, 23) = 4.64 (*p* = .042), but not on Sessions 1 and 10 (*p* values > .09).[Table-anchor tbl4]

Levels of pre-CS responding were low for all three cues (mean overall rate of responding = 3.14 RPM ± 0.20 *SEM*). A Cue × Session ANOVA revealed that pre-CS responding declined over sessions, *F*(9, 207) = 20.48, *p* < .001, η_p_^2^ = .47, 90% CI [.37, .52], but there were no other significant main effects or interactions (*F* values < 1, *p* values > .8).

The rates of responding during the 1–10 s and 31–40 s periods of the reinforced cues can be seen in Figure 7C–7D. The rates of responding were higher for the 10+/40+ cue during both periods. Analysis of the gradients of normalized responding (1–10 s: 10+/40−: *M* = 0.00874, *SEM* = 0.00087; 10+/40+: *M* = 0.00917, *SEM* = 0.00116; 31–40 s: 10+/40−: *M* = −0.00222, *SEM* = 0.00097; 10+/40+: *M* = 0.00110, *SEM* = 0.00058; see Figure 7E–7F) showed significant main effects of cue, *F*(1, 23) = 5.57, *p* = .027, and period, *F*(1, 23) = 94.8, *p* < .001, showing steeper gradients for the 10+/40+ cue than the 10+/40− cue and steeper gradients during the 1–10 s period than the 31–40 s period. The Cue × Period interaction was nonsignificant, *F*(1, 23) = 2.93, *p* = .10.

The results demonstrate that mice are able learn about events that occur after reinforcement, within the same trial. Mice responded more to the cue that was reinforced with two pellets per trial, one after 10 s and one after 40 s, compared to the cue to that was reinforced once after 10 s. This was true for the 10 s period prior to the time of the second reinforcement (31–40 s) and also for the first 10 s of the cues prior to the first reinforcement. These results provide clear evidence that mice are able to effectively process the continued presentation of a cue after reinforcement occurs. This suggests that the lack of difference between cues that were matched for delay of reinforcement but had differing rates of reinforcement in Experiment 3 was not due to insufficient processing of the nonreinforced exposure after the occurrence of reinforcement.

## General Discussion

We previously found that the CS duration effect was abolished if the rate of reinforcement was equated across CSs (Austen et al., 2018), suggesting that the cause of the CS duration effect was sensitivity to reinforcement rate. The present results, however, provided little support for that conclusion. In Experiment 3, when CSs differed in reinforcement rate but were matched for delay of reinforcement, there was no significant difference in rate of conditioned responding. In contrast, when CSs were matched for reinforcement rate, but differed in delay of reinforcement then the CS with a short delay elicited higher rates of responding compared to the CS with a long delay.

These findings contradict rate estimation theory (Gallistel & Gibbon, 2000), which states that the acquisition of conditioned responding reflects calculation of the rate of reinforcement across cumulative exposure to a cue. The model proposes that animals represent and store CS durations and number of reinforcements in memory such that these variables can be used to derive rate information. Because of the calculation of rate occurring across cumulative CS exposure, independent of how the CS exposure is divided into specific trial durations, and independent of the delay of reinforcement within those trials, the model predicts that CSs that differ in reinforcement rate will elicit different rates of responding. The assumption that rate is calculated across cumulative exposure is key to the model’s ability to explain various properties of conditioning such as the contingency effect (Rescorla, 1968), in which the background reinforcement rate (i.e., the rate in the absence of the CS) affects the rate of responding to the CS.

The results are also problematic for a simple associative account of the role of reinforcement rate in learning that assumes that changes in associative strength occur moment by moment rather than trial by trial. Thus, it is possible to derive an account of sensitivity to reinforcement rate by assuming that during periods of CS exposure in which reinforcement occurs there are increments in associative strength, but during periods of CS exposure in which reinforcement does not occur then there are decrements in associative strength. This simply results in a CS gaining associative strength that is proportional to the reinforcement rate. Therefore, CSs may differ in duration and delay of reinforcement, but as long as their reinforcement rate and cumulative duration of exposure are matched then they will gain the same associative strength.

Although rate estimation theory (Gallistel & Gibbon, 2000) proposes that the duration of cumulative CS exposure is the critical variable over which reinforcement rate is calculated, it is tempting to speculate whether other intervals could be used in order to derive estimations of rate that could also account for the results of Experiment 3. For example, rate estimation theory also assumes that animals encode the delay of reinforcement in order to time conditioned responses. If the reinforcement rate simply reflected the inverse of the delay of reinforcement on reinforced trials alone (e.g., Gibbon & Balsam, 1981), rather than the number of reinforcements across the cumulative duration of CS exposure, it would provide a potential account of the results of Experiment 3 in which cues with matched delay of reinforcement but different reinforcement rates elicited the similar levels of responding. In this situation the differences in duration of continued CS exposure after the US would have no effect on reinforcement rate. This account, however, would not explain how matching reinforcement rates over cues that differ in probability of reinforcement per trial leads to matched rates of responding. For nonreinforced trials there is no CS–US interval. This raises the issue of what temporal information is encoded on nonreinforced trials. Models such as rate estimation theory (Gallistel & Gibbon, 2000) and the application of scalar expectancy theory to acquisition of conditioned responding (Gibbon & Balsam, 1981) assume that nonreinforced trials simply add to the cumulative CS exposure and therefore reduce estimations of rate or temporal expectancy of reinforcement in the same manner as increasing the CS duration for reinforced trials. Although it is possible that variables other than cumulative exposure may account for the present results, such variables are unlikely to account for findings such as the contingency effect (Dweck & Wagner, 1970; Murphy & Baker, 2004; Rescorla, 1968) in which the reinforcement rate over cumulative exposure does appear to be the crucial factor. Thus, it is not readily obvious what other variables may be encoded which provide a satisfactory account of the results.

It is clear from Experiment 3 that extending the duration of CS exposure beyond the presentation of the US failed to extinguish conditioned responding. This was true even when there was an extra 150 s of CS exposure beyond the time at which reinforcement occurred compared to a cue for which there was an extra 30 s after reinforcement. As mentioned above, this is problematic for time-sensitive associative accounts of learning that assume that changes in learning occur moment by moment, and, therefore, nonreinforced CS exposure will lead to extinction of excitatory learning regardless of when it is presented within a trial. The lack of extinction, however, may be accounted for by a number of assumptions. For example, if it is assumed that the associability of the CS decreases within a trial due to short-term habituation (Wagner, 1981), then it is possible that the nonreinforced CS exposure after reinforcement was not sufficient for extinction of learning to occur. Alternatively, if a componential view of CS representations is assumed such that a stimulus consists of a series of temporally ordered microstimuli (e.g., Ludvig et al., 2012; Sutton & Barto, 1981, 1990; Vogel et al., 2003), it is possible that the continued CS exposure after the US had no effect because the nature of the CS representation during those periods was different to its representation prior to reinforcement. Temporal difference learning models have appealed to changes in the nature of stimulus representations within a trial in order to explain various aspects of timing behavior (e.g., Williams, Todd, Chubala, & Ludvig, 2017). In the case of the results in Experiment 3, there would have had to be little or no generalization of learning between elements of the CS representation that were processed prior to reinforcement and after reinforcement. Temporal difference learning models would predict this to be the case when it is assumed that a stimulus is represented as a complete serial compound in which each temporally activated element is entirely distinct (Sutton & Barto, 1990). The complete serial compound assumption, however, leads to incorrect, and perhaps unrealistic, predictions about the precision of timing in terms of behavior and neural correlates of prediction error learning (Gershman, Moustafa, & Ludvig, 2014; Ludvig et al., 2012).

A reason for doubting explanations of the failure to observe extinction in Experiment 3 in terms of reduced associability or changes in the nature of the stimulus representation are the results of Experiment 4. In that experiment, we found that mice could learn about reinforcement that occurred after an initial reinforcement within a trial, suggesting that, at the least, the associability of the stimulus at that time point was sufficient for learning to occur, albeit the learning was excitatory rather than reflecting extinction. In addition, we found that mice responded more during the first 10 s of the cue that received two reinforcements within a trial compared to the cue that was reinforced at the first time point, but not the second. Therefore, learning about the cue prior to the second reinforcement generalized to the first 10 s period of the cue prior to the first reinforcement. This would suggest that, at the least, there was commonality between the nature of the CS representation at different time points within the CS. Temporal difference models would only be able to account for the data by making extreme assumptions about the changes in the nature of the CS representation within a trial.

The experiments presented here sought to identify the role of delay of reinforcement in the CS duration effect. A complication in trying to dissociate the effects of delay and rate of reinforcement in the CS duration effect was that attempts to control one factor while manipulating another ran the risk of introducing new confounds. Thus, in Experiment 2 the manipulation of rate, by comparing a short, delay conditioned cue with a long, simultaneously conditioned cue, led to confounds in temporal contiguity. Experiment 3 did, however, provide clear results demonstrating an effect of delay of reinforcement, but not rate of reinforcement. This dissociation was revealed only by comparing conditioned responding to cues for which reinforcement occurred during the CS presentation. It is also important to note that the results were found with female mice using an appetitive Pavlovian magazine approach procedure. Therefore, future work will need to establish the generality of these findings to other species and conditioning paradigms.

In conclusion, it is not clear how delay of reinforcement alone can account for why matching reinforcement rate between cues of different durations and different probabilities of reinforcement per trial leads to matched response rates. The present results do, however, suggest that delay of reinforcement may be more important than reinforcement rate in particular circumstances. The results provide a challenge to accounts of learning that assume that the cumulative exposure to a cue is a critical variable in determining response rates.

## Figures and Tables

**Table 1 tbl1:** Mean (SEM) Responses per Minute (RPM) During the Nonreinfored Cues in Experiment 1

Group	CS duration (s)	Session											
		1	2	3	4	5	6	7	8	9	10	11	12
Fixed	10	2.2 (.4)	4.8 (.9)	9.0 (1.2)	7.3 (1.7)	3.8 (.6)	3.1 (.6)	3.5 (.8)	2.5 (.6)	1.9 (.6)	1.9 (.5)	1.5 (.4)	1.9 (.4)
	40	2.5 (.3)	3.9 (.6)	5.4 (.8)	3.6 (.6)	1.8 (.3)	1.4 (.3)	1.4 (.2)	1.7 (.3)	1.3 (.2)	1.1 (.2)	1.1 (.2)	1.3 (.3)
Variable	10	2.5 (.5)	7.4 (1.3)	7.3 (1.4)	4.3 (.7)	3.4 (.6)	2.7 (.3)	2.7 (.5)	1.5 (.3)	1.2 (.3)	1.5 (.2)	1.1 (.2)	1.0 (.3)
	40	3.4 (.4)	5.7 (1.1)	4.6 (.8)	2.8 (.4)	1.5 (.3)	1.4 (.2)	1.0 (.2)	.8 (.1)	1.0 (.2)	.7 (.1)	.7 (.1)	.8 (.1)
*Note*. CS = conditioned stimulus.													

**Table 2 tbl2:** Mean (SEM) Responses per Minute (RPM) During the Nonreinforced Cues in Experiments 2a and 2b

Experiment	Condition	Session											
		1	2	3	4	5	6	7	8	9	10	11	12
2a	40/10+	5.4 (1.1)	9.6 (1.6)	11.7 (2.1)	7.2 (1.4)	2.3 (.6)	4.5 (1.4)	5.7 (1.4)	2.9 (.6)	2.9 (.7)	2.0 (.6)	3.7 (.7)	2.6 (.6)
	40/40+	4.8 (.9)	6.7 (1.3)	6.7 (1.1)	3.5 (1.1)	1.8 (.4)	2.4 (.6)	3.6 (.8)	2.0 (.4)	1.3 (.2)	1.8 (.4)	1.8 (.4)	2.2 (.5)
2b	10/10+	4.9 (.9)	8.6 (1.6)	7.6 (2.1)	4.5 (1.1)	3.9 (1.5)	2.5 (.6)	3.9 (.8)	1.9 (.5)	1.9 (.7)	1.6 (.8)	1.6 (.6)	1.4 (.5)
	40/10+	4.7 (.9)	6.9 (1.5)	7.4 (2.0)	4.9 (1.3)	3.7 (1.1)	1.5 (.4)	3.4 (1.0)	2.4 (.6)	1.6 (.6)	2.1 (.7)	1.1 (.4)	2.1 (.5)

**Table 3 tbl3:** Mean (SEM) Responses per Minute (RPM) During the Nonreinforced Cues in Experiment 3

Group	Cue duration (s)	Session											
		1	2	3	4	5	6	7	8	9	10	11	12
70/10+	40	2.9 (.6)	4.2 (1.0)	6.4 (1.7)	5.6 (1.0)	4.9 (1.0)	4.8 (1.2)	4.0 (.9)	1.8 (.7)	2.3 (.5)	2.1 (.7)	2.6 (.4)	1.9 (.6)
	70	3.1 (.7)	4.7 (.8)	5.4 (1.1)	5.6 (1.4)	3.7 (.9)	2.6 (.7)	2.1 (1.0)	3.1 (1.0)	2.8 (.8)	1.7 (.4)	2.1 (.6)	2.5 (.8)
70/40+	40	2.7 (.6)	6.0 (1.3)	6.4 (1.3)	5.1 (1.4)	5.0 (1.2)	5.1 (.8)	3.6 (.9)	3.1 (.7)	2.3 (.7)	2.9 (.9)	2.3 (.5)	2.9 (.6)
	70	3.1 (.5)	4.4 (.6)	3.8 (.6)	2.4 (.5)	1.8 (.2)	1.6 (.2)	1.8 (.4)	1.1 (.3)	1.2 (.3)	1.3 (.3)	1.1 (.2)	1.2 (.2)
160/10+	40	3.1 (.5)	6.4 (1.2)	10.3 (2.3)	7.6 (1.1)	4.1 (1.0)	4.8 (1.2)	3.8 (.9)	2.5 (.4)	1.4 (.5)	.9 (.3)	1.8 (.7)	1.1 (.4)
	160	5.1 (.9)	5.2 (.7)	5.8 (1.0)	5.4 (1.6)	4.3 (1.3)	3.1 (.5)	2.0 (.7)	2.8 (.6)	1.3 (.4)	2.6 (.9)	1.9 (.7)	2.9 (.7)
160/40+	40	2.8 (.5)	5.0 (1.0)	7.3 (1.6)	8.5 (2.2)	7.6 (1.3)	5.6 (.9)	5.6 (1.2)	3.1 (.8)	3.3 (.9)	1.9 (.6)	3.1 (1.0)	2.3 (.5)
	160	3.0 (.4)	4.3 (.5)	3.5 (.6)	2.9 (.8)	1.8 (.3)	2.2 (.2)	1.9 (.5)	1.4 (.3)	1.4 (.3)	1.1 (.2)	2.0 (.4)	1.8 (.3)

**Table 4 tbl4:** Mean (SEM) Responses per Minute (RPM) During the Nonreinforced Cue in Experiment 4

CS period	Session									
	1	2	3	4	5	6	7	8	9	10
1–10 s	4.3 (.5)	10.3 (1.1)	9.7 (1.5)	8.4 (1.5)	5.1 (.7)	3.6 (.6)	4.5 (1.1)	3.2 (.5)	4.2 (.7)	1.8 (.3)
31–40 s	3.4 (.4)	6.9 (.7)	3.1 (.5)	2.8 (.5)	1.8 (.4)	1.5 (.3)	1.8 (.3)	1.8 (.3)	2.3 (.7)	1.7 (.3)
*Note*. CS = conditioned stimulus.
